# Breakthrough Infections of E484K-Harboring SARS-CoV-2 Delta Variant, Lombardy, Italy

**DOI:** 10.3201/eid2712.211792

**Published:** 2021-12

**Authors:** Andreina Baj, Federica Novazzi, Renee Pasciuta, Angelo Genoni, Francesca Drago Ferrante, Marilena Valli, Michele Partenope, Rosalia Tripiciano, Andrea Ciserchia, Giuseppe Catanoso, Daniele Focosi, Fabrizio Maggi

**Affiliations:** ASST SetteLaghi, Varese, Italy (A. Baj, F. Novazzi, R. Pasciuta, F. Drago Ferrante, F. Maggi);; University of Insubria, Varese (A. Baj, A. Genoni, F. Maggi); St. Anna Hospital, Como, Italy (M. Valli, M. Partenope);; ATS Insubria, Varese (R. Tripiciano, A. Ciserchia, G. Catanoso);; Pisa University Hospital, Pisa, Italy (D. Focosi)

**Keywords:** SARS-CoV-2, COVID-19, coronavirus disease, severe acute respiratory syndrome coronavirus 2, viruses, respiratory infections, zoonoses, variant of concern, delta variant, mutation of concern, B.1.617.2, E484K, Italy

## Abstract

The Delta variant of concern of severe acute respiratory syndrome coronavirus 2 is dominant worldwide. We report a case cluster caused by Delta sublineage B.1.617.2 harboring the mutation E484K in Italy during July 11–July 29, 2021. This mutation appears to affect immune response and vaccine efficacy; monitoring its appearance is urgent.

Since the beginning of 2021, a severe acute respiratory syndrome coronavirus 2 (SARS-CoV-2) variant originally described in India has become the predominant circulating variant of the coronavirus disease pandemic. This variant of concern (VOC) was renamed Delta by the World Health Organization and consists to date of 5 different sublineages (B.1.617.2, AY.1, AY.2, AY.3, and AY.3.1, according to PANGOLIN phylogeny) that share T478K and L452R as the main mutations of concern (MOCs) within the spike protein. B.1.617.2 (also known as VUI-21APR-02) is by far the most represented Delta sublineage. None of the 5 sublineages are to date characterized by the occurrence of the other MOC E484K, which causes resistance to monoclonal antibodies and reduced vaccine efficacy. However, given the widespread convergent evolution of the spike protein observed across clades, the occurrence of MOC E484K and its widespread circulation is largely expected. A clade simultaneously harboring all such MOCs is likely to be of extreme concern because of theoretical increased immune escape. We report a cluster of B.1.617.2 and E484K occurring in Lombardy, Italy. All cases were first tested by real-time reverse transcription PCR and, if positive, sequenced as previously reported (*1*).

On July 11, 2021, a 41-year-old man from a small village in northern Lombardy (vaccinated with BNT162b2 [Pfizer-BioNTech, https://www.pfizer.com] on June 12 and July 12) began experiencing cough, fever, and malaise; a nasopharyngeal swab specimen tested positive on July 14 by the SARS-CoV-2 Variants Elite MGB Kit (EliTech Group, https://www.elitechgroup.com); cycle threshold (C_t_) was 21 for open reading frame (ORF) 1ab gene and 21 for the nucleocapsid (N) gene. He fully recovered without need for hospital admission; whole-genome sequencing confirmed B.1.617.2 that harbored E484K. His 80-year-old mother (vaccinated with mRNA-1273 [Moderna, https://www.modernatx.com] on April 9 and May 7) experienced fatigue, headache, myalgia, and dyspnea beginning July 17 and tested positive on July 24 (C_t_ 22 for ORF1ab gene and C_t_ 21 for N gene). She likely further infected (while playing cards) a 77-year-old man (vaccinated with BNT162b2 on April 26 and May 17) who began experiencing fever July 21 and tested positive on July 23 (C_t_ 20 for ORF1ab gene and C_t_ 19 for N gene) and an 83-year-old woman (vaccinated with BNT162b2 on April 3 and April 24) who experienced fever, fatigue, ageusia, and anosmia beginning July 21 and tested positive July 24 (C_t_ 18 for both genes). None required hospital admission. An unrelated patient from the same village, an 81-year-old woman (vaccinated with mRNA-1273 on May 7 and June 9), experienced dyspnea, fever, myalgia, and fatigue beginning July 24. On July 29, she tested positive for SARS-CoV-2 RNA (C_t_ 23 for ORF1ab gene and C_t_ 21 for N gene), and she was admitted to the hospital. All sequences obtained in this study have been deposited into GISAID (https://www.gisaid.org; accession nos. EPI_ISL_3462078, EPI_ISL_3462074, EPI_ISL_3462072, EPI_ISL_346208).

E484K is the hallmark MOC of VOCs Beta and Gamma, in addition to having been reported in a minor sublineage of VOC Alpha, in variants of interest Eta and Iota, and at frequencies >50% in 38 more strains. E484K causes resistance to many class 2 RBD-directed antibodies (*2*), including bamlanivimab (*3*). The most potent mRNA vaccine–elicited monoclonal antibodies were >10-fold less effective against pseudotyped viruses carrying the E484K mutation (Z. Wang et al., unpub. data, https://www.biorxiv.org/content/10.1101/2021.01.15.426911v2). As of August 12, 2021, GISAID reported E484K in 52 of 408,781 B.1.617.2 sequences, 2 of 549 AY.1 sequences, and 32 of 19,996 AY.3 (Delta) sequences; none of these reports were in Italy. E484K has been additionally reported in 1 of 6,011 B.1.617.1 (Kappa variant) sequences (*4*).

Nasopharyngeal swab specimens positive for the Delta variant have ≈4-fold higher viral loads than non-VOC or Alpha variants (C. von Wintersdorff et al., unpub. data, https://www.researchsquare.com/article/rs-762916/v1) and a shorter incubation time of 4 days (B. Li et al., unpub. data, https://www.medrxiv.org/content/10.1101/2021.07.07.21260122v1). It is resistant to REGN10933 (T. Tada et al., unpub. data, https://www.biorxiv.org/content/10.1101/2021.07.19.452771v3) and bamlanivimab (M. Hoffman et al., unpub. data, https://www.biorxiv.org/content/10.1101/2021.05.04.442663v1; P. Arora et al., unpub. data, https://www.biorxiv.org/content/10.1101/2021.06.23.449568v1), whereas neutralization by antibodies derived from cyclic citrullinated peptide, BNT162b2, mRNA-1273, and Ad26.COV2.S are reduced by 3–5-fold (T. Tada et al., unpub. data).

E484K mutation represents a critical evolutionary event that leads to immune escape, although its consequences on viral fitness are unclear. Surveillance by genome sequencing should be maintained (T. Farinholt et al., unpub. data, https://www.medrxiv.org/content/10.1101/2021.06.28.21258780v4).
